# Review of Associations between Built Environment Characteristics and Severe Acute Respiratory Syndrome Coronavirus 2 Infection Risk

**DOI:** 10.3390/ijerph18147561

**Published:** 2021-07-15

**Authors:** Jingjing Wang, Xueying Wu, Ruoyu Wang, Dongsheng He, Dongying Li, Linchuan Yang, Yiyang Yang, Yi Lu

**Affiliations:** 1 Department of Architecture and Civil Engineering, City University of Hong Kong, Kowloon Tong, Hong Kong 999077, China; jwang586-c@my.cityu.edu.hk (J.W.); xueyingwu8-c@my.cityu.edu.hk (X.W.); 2 School of Urban Design, Wuhan University, Wuhan 430072, China; 3 Institute of Geography, School of GeoSciences, University of Edinburgh, Edinburgh EH8 9XP, UK; R.Wang-54@sms.ed.ac.uk; 4 Department of Architecture, University of Cambridge, Cambridge CB2 1PX, UK; hedsh3@mail2.sysu.edu.cn; 5 Department of Landscape Architecture & Urban Planning, Texas A&M University, College Station, TX 77843, USA; dli@arch.tamu.edu; 6 Department of Urban and Rural Planning, Southwest Jiaotong University, Chengdu 610031, China; yanglc0125@swjtu.edu.cn; 7 City University of Hong Kong Shenzhen Research Institute, Shenzhen 518057, China

**Keywords:** SARS-CoV-2, COVID-19, built environment

## Abstract

The coronavirus disease 2019 pandemic has stimulated intensive research interest in its transmission pathways and infection factors, e.g., socioeconomic and demographic characteristics, climatology, baseline health conditions or pre-existing diseases, and government policies. Meanwhile, some empirical studies suggested that built environment attributes may be associated with the transmission mechanism and infection risk of severe acute respiratory syndrome coronavirus 2 (SARS-CoV-2). However, no review has been conducted to explore the effect of built environment characteristics on the infection risk. This research gap prevents government officials and urban planners from creating effective urban design guidelines to contain SARS-CoV-2 infections and face future pandemic challenges. This review summarizes evidence from 25 empirical studies and provides an overview of the effect of built environment on SARS-CoV-2 infection risk. Virus infection risk was positively associated with the density of commercial facilities, roads, and schools and with public transit accessibility, whereas it was negatively associated with the availability of green spaces. This review recommends several directions for future studies, namely using longitudinal research design and individual-level data, considering multilevel factors and extending to diversified geographic areas.

## 1. Introduction

### 1.1. Coronavirus Disease 2019

It has been more than one year since the outbreak of coronavirus disease 2019 (COVID-19) in December 2019, and it has spread to most countries and regions worldwide. The outbreak was announced as a public health emergency on 30 January 2020 and then a global pandemic on 11 March 2020 by World Health Organization (WHO) [1]. As of 21 February 2021, more than 110.74 million cases have been confirmed globally, including over 2.45 million deaths [2]. The COVID-19 pandemic has become one of the most catastrophic global health crises in the last several decades.

The three major transmission mechanisms of severe acute respiratory syndrome coronavirus 2 (SARS-CoV-2) are large droplet transmission, aerosol transmission, and fomite transmission [3,4]. Although many social distancing measures, such as limiting large group gathering and mandatory mask-wearing requirement have been enforced and several vaccines have been developed, there is no effective treatment to cure infected individuals. COVID-19 has significantly changed daily life and challenged the global public health system and social economic development [5,6].

### 1.2. Major Factors Associated with SARS-CoV-2 Infection

The COVID-19 pandemic has stimulated intensive research interest of scholars from various disciplines in its transmission pathways and infection factors. Reviews have identified several critical factors associated with SARS-CoV-2 transmission and infection, including socioeconomic and demographic characteristics (e.g., gender, ethnicity, age, and household income) [7,8,9], climatology parameters (e.g., temperature, humidity, wind, and air pollution) [10,11,12], baseline health conditions or pre-existing diseases (e.g., kidney and cardiovascular diseases) [13], and government policies (e.g., social distancing and lockdowns) [14,15].

Built environment can be defined as human-made surroundings, which provide space and place for human activity [16]. As a key sphere of socioecological framework, built environment factors significantly affect long-term health outcomes, and such effects have been identified across different social and urban contexts [17,18]. Evidence also supports that a well-designed built environment can improve human health via several major pathways, e.g., promoting physical activity, reducing stress, increasing social contacts, and reducing pernicious environmental exposures (e.g., air pollution, sanitation, and noise) [19,20,21]. Several built environment factors that influence health have been identified in previous reviews [19,22,23,24]. These factors can be summarized in a five-dimensional (5D) model framework including density, diversity, design, destination accessibility, and distance to transit [22].

Circumstantial evidence supports that built environment characteristics may be related to the transmission of coronavirus infection. In previous existing literature, a large number of studies have revealed the relationship between built environment and the transmission mechanism and infection risk of SARS-CoV-2 because the built environment affects how people move around and the human-to-human contact in outdoor and indoor environments [25,26,27]. For example, a higher density of service facilities (e.g., commercial facilities, schools, hospitals) may increase the risk of close contact, thus leading to the person-to-person SARS-CoV-2 transmission. In addition, public transit passengers may have high infection risk due to prolonged virus exposure within the enclosed carriages. However, evidence related to the effect of special built environment characteristics on the SARS-CoV-2 infection risk is inconclusive. For instance, people who lived in high-density areas may have more social contacts in their daily lives and thus a high risk of infection [25]. Whereas cities and nations with a higher population density were found to implement stricter regulations, which effectively alleviated the spread of the virus [28]. No review has summarized the role of the built environment in the COVID-19 pandemic. This gap in knowledge about built environment characteristics and to what extent they affect SARS-CoV-2 infection should be addressed, as a lack of knowledge may prevent government officials and urban planners from creating effective guidelines and urban environments to contain SARS-CoV-2 infections and face future pandemic challenges.

### 1.3. Our Contributions

Given the research gaps discussed in Section 1.2, this review aimed at summarizing the existing evidence and providing an overview of the effect of built environment on SARS-CoV-2 infection during the COVID-19 pandemic. First, we identified the critical built environment factors that affect SARS-CoV-2 infection by comprehensively reviewing empirical studies on this topic. Second, we explored the potential mechanisms by which the built environment characteristics affect SARS-CoV-2 infection. Our study may help to identify high-risk urban areas and thus develop effective strategies to reduce SARS-CoV-2 infection via targeted interventions. Our study may also contribute to providing urban planning guidelines to cope with future pandemics. 

## 2. Methods

This research was conducted based on the Preferred Reporting Items for Systematic Reviews and Meta-Analyses (PRISMA) criteria [29]. We delivered a systematic search using meta-database, including PubMed, Scopus, Web of Science, and preprint servers (medRxiv, bioRxiv, and arXiv) from inception to 3 June 2021. The search adopted the following keywords in the article title or abstract for relevant studies: (“built environment” OR “urban environment” OR “neighborhood” OR “neighborhood” OR “physical environment” OR “land use” OR “proximity” OR “distance to destination” OR “population density” OR “urban density” OR “building density” OR “green space” OR “greenery” OR “parks”) AND (“COVID-19” OR “SARS-CoV-2” OR “novel coronavirus” OR “2019-nCoV” OR “nCoV” OR “novel beta-CoV” OR “novel betacoronavirus”). 

The following three selection criteria were used to identify the eligibility of the retrieved studies for inclusion in the current review:

1. Examined the association between COVID-19 and certain aspects of the built environment;
2. Were written in English; and
3. Were not letters, notes, opinions, commentaries, or reviews.

**Figure 1:**
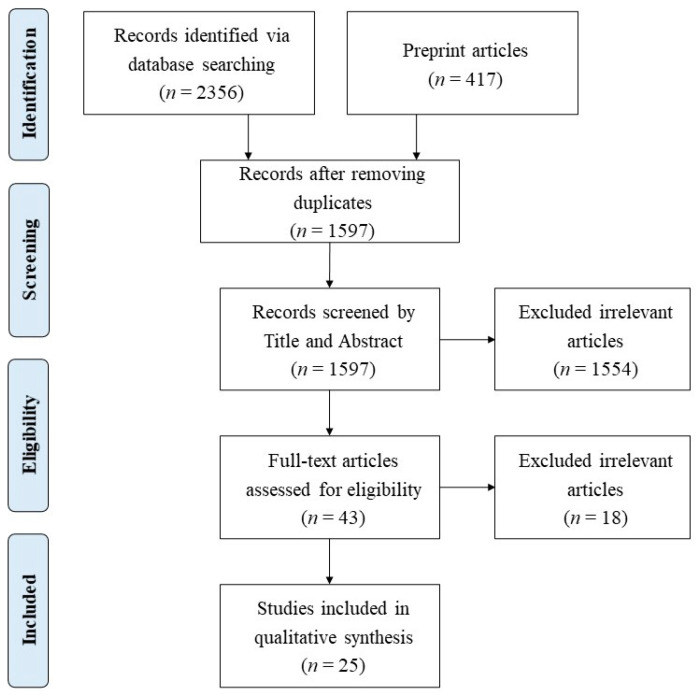
The workflow diagram based on Preferred Reporting Items for Systematic Reviews and Meta-Analyses study selection method.

We first screened the title and abstracts to exclude irrelevant studies and then evaluated the full-text articles to remove those that failed to meet the above criteria. Our final review database contained 25 eligible full-text articles. The PRISMA diagram is displayed in Figure 1.

## 3. Results

### 3.1. Study Characteristics

We reviewed 25 studies included in qualitative synthesis. Appendix A summarizes the following characteristics from each included sample study: authors, country, research design, geography unit, sample size, COVID-19 metrics, built environment metrics, data analysis method, and major results. “COVID-19 metrics” refers to the measurements related to SARS-CoV-2 infection, such as the case number and incidence. The top two studied countries were China and the United States with 10 studies, respectively; four studies were conducted in Europe and South Asia, including England (*n* = 2), Germany (*n* = 1), and Bangladesh (*n* = 1); and one study was conducted in multiple countries. Except for one longitudinal study, all studies followed a cross-sectional study design. Most of the studies were performed at the nationwide or city level, whereas some studies chose a smaller geographical unit, such as the zip code or neighbourhood.

### 3.2. Built Environment Metrics

**Figure 2:**
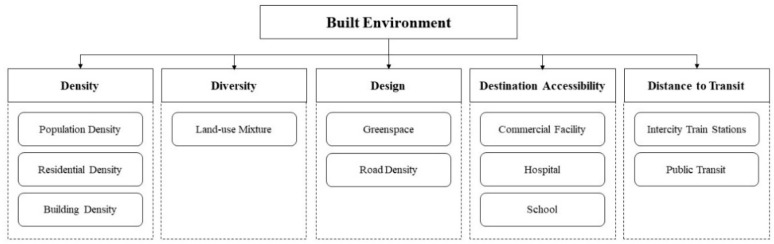
The framework of built environment metrics in current review.

The built environment metrics assessed by these studies included urban density, land-use mixture, connectivity, accessibility to public transit, accessibility to destinations, availability of green space, and other surrogate measures, which are largely in line with the 5D framework as Figure 2. Considerable heterogeneity within included studies was found in terms of the selection and definition of built environment metrics, which hindered the pool effects estimation. In brief, nine built environment metrics were assessed in the studies. There were also various measuring methods for defining composite indexes, for example, urban density and land-use mixture. The most widely studied built environment characteristics, which were included in at least six studies, were urban density (including population density, building density, and residential density), commercial facility and hospital density, availability of green space, and accessibility to public transit. The data source of these metrics was various, including census data, remote sensing data, open-source urban land-use data, Google Street View images, and other public records.

### 3.3. COVID-19 Metrics

The COVID-19 metrics were measured in four aspects, namely the number of COVID-19 confirmed cases (*n* = 9), COVID-19 incidence (the ratio of COVID-19 cases divided by population size) (*n* = 14), COVID-19 cluster size (the number of groups of COVID-19 cases) (*n* = 1), and basic reproductive number (a function to describe the virus transmissibility among population) (*n* = 1). The confirmed COVID-19 case data in these studies were mainly obtained from government statistics.

### 3.4. Associations between the Built Environment and SARS-CoV-2 Infection

Table 1 lists the directions of associations between each built environment variable and SARS-CoV-2 infection in all 25 selected studies (see more details in Table A1). A study might report multiple associations for the same built environment–infection association because of different statistical tests and adjusted covariates. The association directions were coded as “+,” “−,” or “0;” “+” denotes a statistically significant positive association; while “−” represents a statistically significant negative association; and “0” indicates no significant association. Table 2 summarizes the number of each association from all studies.

Following previous reviews [54,55], a built environment characteristic will be identified as having strong evidence if the amount of positive associations was greater than or equal to the sum of its negative or inconclusive associations (“+” ≥ “−/0”) or amount of negative associations was greater than or equal to the sum of its positive or inconclusive associations (“−” ≥ “+/0”). As shown in Table 2, there was strong evidence for positive associations of virus infection risk with commercial facility density (11 “+” vs. 2 “−/0;” 84.6%), elementary and middle school density (2 “+” vs. 1 “−/0;” 66.7%), accessibility to public transit (3 “+” vs. 3 “−/0;” 50.0%), and road density (2 “+” vs. 2 “−/0;” 50.0%). There was also strong evidence for a negative association between infection risk and availability of green space (4 “−” vs. 3 “+/0;” 57.1%). The evidence for associations of infection risk with other built environment factors, including urban density, hospital density, and accessibility to intercity train stations, was weak.

## 4. Discussion

Twenty-five studies were selected in the current review. Although the sample size was limited, our review still provides some conclusions and insights into the potential influences of built environment characteristics on SARS-CoV-2 infection risk. Overall, the commercial facility density, school density, accessibility to public transit, and road density were positively associated with SARS-CoV-2 infection, whereas availability of green space was negatively associated with it. Evidence for some important built environment factors was inconclusive, such as urban density and land-use mixture, which were proven to be related to long-term active travel behaviours and health outcomes of urban residents [56,57,58].

### 4.1. Major Findings

#### 4.1.1. Commercial Facility Density

In this review, the strongest evidence was found for the association between commercial facilities and SARS-CoV-2 infection risk. Eleven out of 13 studies (84.6%) reported positive associations. One plausible explanation is that people who lived in neighbourhoods with more commercial destinations and services are more likely to use these facilities [21,59,60], therefore their risk of exposure to the virus increased [30,38]. Besides, in some recreation and service facilities, such as restaurants, hotels, and bars, people tend to take off their masks to talk, drink, and dine [38]. Furthermore, a large number of the commercial locations are designed as indoor spaces with inadequate ventilation, which can easily become high-risk places because the spread of the virus is intensified in confined spaces [41]. Hence, urban areas with intensive commercial facilities may be confronted with a higher virus infection risk. Some social distancing measures, for example, closure of unnecessary commercial destinations and/or controlling the number of people in such destinations, are needed. 

#### 4.1.2. School Density 

Three studies investigated the association between school density (e.g., schools of different categories (elementary and middle schools)) and infected cases. Two studies in China showed a positive association [38,47], whereas one study in the U.S. revealed an insignificant association [33]. The potential mechanism linking school density and infection risk is that many teachers and students gather in classrooms and frequently interact with each other at a short distance in class and in after-class activities. In this setting, long and intimate contact in a closed environment may significantly increase virus transmission [61].

Given that many schools have been conducting online teaching rather than face-to-face teaching, the teaching mode may have also modified the observed results. Thus, more studies are needed to explore the relationship between school density, teaching mode, and infection risk. School density may also be a proxy for other constructs. For instance, the number of schools may be a measure of population density or socioeconomic status (SES) of an area because public schools are funded by local tax revenues in some countries, e.g., China and U.S. [33]. Although the infection risk is more pronounced in areas with a high school density, the causal relationship is still uncertain. Therefore, the potential influence of school density needs to be further investigated.

#### 4.1.3. Road Density

Two out of four studies showed that road density is positively associated with virus infection [36,47]. A neighbourhood with a higher road density may have higher mobility and pedestrian activities [62] and consequently a higher risk of virus exposure and transmission. However, the results of two studies conducted in Bangladesh and China showed that road density had no significant effect on SARS-CoV-2 incidence [41,45]. This might have been due to the local social restrictions established to contain the rapid spread of COVID-19. For example, Wuhan, China, implemented a total lockdown policy during the data collection period, which sharply decreased the traffic flow and pedestrian activities [41]. 

#### 4.1.4. Accessibility to Public Transit

Three out of six studies showed positive associations between accessibility to public transit and virus infection [37,38,42]. Public transit conveys many passengers for daily commuting or other activities in confined and often crowded settings. Given the potential for virus exposure among public transit passengers, the risk of virus transmission is substantial [63].

However, one study revealed a more complex relationship and found that the impact of public transit on the prevalence of COVID-19 was significant only when social distancing measures were relaxed [31]. People may mitigate the potential virus infection risk associated with public transit by using private vehicles, staying home, or wearing masks. The demand for public transit significantly decreased during the pandemic [64]. Furthermore, many cities implemented compulsory measures for public transit passengers (e.g., wearing masks and maintaining social distancing) [65]. Thus, the influence of accessibility to public transit may vary according to different pandemic stages, social distancing measures, and social contexts.

#### 4.1.5. Availability of Green Space 

Four out of seven studies showed the negative association between the availability of green space and COVID-19 incidence [42,49,66,67]. There are two possible explanations for this result. First, it is widely recognized that green space can promote long-term physical and mental health [67,68] by supporting physical activity and providing stress relief, which may help to boost the immune system against the virus. Second, air pollution may exacerbate the SARS-CoV-2 infection risk [69], yet green space can reduce exposure to air pollution, thereby decreasing the virus infection risk [67]. Other studies also found that green space usage increased during the pandemic [70], and the availability of green space decreases racial disparity in virus infection rates [71]. However, two studies suggested a positive association between green space and virus infection risk [48,52]. Some researchers believe that green spaces may promote close contact and increase infection risk, although the outdoor infection risk is low. People may also be infected when using public fitness facilities and public toilets in green spaces, which involve physical touch. Overall, with adequate precautions (e.g., controlling the number of users in green spaces, social distancing, and hygiene), the provision of green space may be an effective urban design strategy to face the challenge from the COVID-19 pandemic and future pandemic crises. 

#### 4.1.6. Urban Density 

Urban density, which was often assessed by population density, building density, or residential density, was the most intensively investigated factor when discovering the relationship between built environment characteristics and COVID-19 incidence. One of the potential explanations is that urban density is commonly positively associated with the rates of infection during pandemics, and population-related data are easier to obtain [34]. 

The evidence for urban density is not conclusive. Only eight out of 24 (33.3%) studies found that urban density was positively associated with COVID-19 incidence. This may account for that in high-density areas, e.g., large cities or urban centres, people may have more social contacts, which may lead to a higher infection risk compared with that in low-density areas. However, five studies reported a negative association and 11 reported an insignificant association. Such mixed results may be explained by three reasons. First, some affluent and high-density cities, especially those in developed countries, have high-quality and accessible health care systems [34,72]. Second, the social distancing policies in high-density areas may be taken more seriously and managed strictly by the government and urban residents, thereby leading to a lower infection rate in these areas [28]. Third, the modifiable areal unit problem [72], i.e., using different spatial scales of analytical units, such as a community, town, county, or census tract, when calculating the area-based urban density or infection rate, may lead to different results in different studies [72]. 

Therefore, although urban density is arguably the most important built environment characteristic and planning parameter, little is known about its effect on SARS-CoV-2 infection. More studies with rigorous research designs are needed on this topic.

#### 4.1.7. Hospital Density

The hospital density results were inconsistent. Three out of eight studies reported positive associations between hospital density and COVID-19 incidence [38,41,47]. Hospitals may be a hot spot for virus transmission because of close person-to-person contact and crowded indoor environments [73]. Many people, including patients, visitors, and healthcare staff, were infected in hospitals due to a lack of understanding about SARS-CoV-2 infection and a lack of appropriate protection during the initial COVID-19 outbreak in China [41]. However, one study in China showed inverse associations between hospital density and COVID-19 incidence [48]. The authors argued that patients can be scattered when there are more hospitals in one area, which may reduce the risk of transmission. Four studies found no significant relationship between hospital density and COVID-19 incidence [30,31,32]. Overall, the inconsistent evidence from these studies indicated that the effect of hospital density on COVID-19 is confounded by other factors, such as the health conditions of residents. Hospital density may also be a proxy for other latent constructs, such as the SES of an area or medical care conditions.

### 4.2. Recommendations for Future Studies

Future studies should address the following four limitations identified in this review.

First, all studies covered in this review, except for one, used a cross-sectional study design, which prevented us from establishing any causality. A major issue in cross-sectional research design is the residential self-selection bias [19,74]. This means that people who have a predisposition for physical activity and a healthy lifestyle may prefer to choose neighbourhoods with space or facilities supporting physical activity and healthy lifestyles [19]. With such bias, observed built environment–health associations may be explained by potential individual attitudes and preferences for physical activity and healthy lifestyles and failed to infer a true causal relationship. Thus, the impact of built environment characteristics on health outcomes, including SARS-CoV-2 infection risk, can be overrated in cross-sectional studies design as well. More controlled and longitudinal studies are needed to determine robust and long-term associations between built environment characteristics and COVID-19 incidence [44,75]. The availability of a suitable control group in a prospective longitudinal study will help us to establish a natural experiment by ruling out the self-selection bias [75]. 

Second, owing to the limited COVID-19 pandemic information released, the incidence and infection data were often offered as aggregated at the county or city level. All of the selected studies in this review also measured built environment characteristics at the county or city level. Such aggregated data are subject to ecological fallacy, which means that we cannot infer the outcome of individuals based on group information [76]. In addition, there are notable variations in built environment exposure for people living in the same county or city [60]. In future research, measuring of individual infection risk and corresponding individual-level built environment exposure is warranted to address this limitation.

Third, besides the built environment, social, cultural, and behavioural factors may also influence the spread of COVID-19, such as social norms, social distancing policies, individual mobility, and behaviours [49,66,67]. Such factors were often neglected in recent studies of built environment–infection associations. According to the socioecological framework, SARS-CoV-2 infection risk is affected by multilevel factors, such as individual (e.g., sex, age, and attitudes), behavioural (e.g., mobility and social interaction), social environment (e.g., family and friends), built environment, natural environment (air pollution, humid, and temperature), community (e.g., norms of wearing masks), and public policy (e.g., social distancing measures) factors. These multilevel factors may interact with each other and make the impact of the built environment on infection risk more complex. Therefore, it is necessary to control such covariates or to investigate the interactions between the built environment and the social environment in future studies. 

Fourth, except for one multinational study, the remain studies selected in the current review were conducted in only five countries. Given the impact of the COVID-19 pandemic in almost all countries and the significant variations among the social and built environments across countries, additional studies covering more countries are required to allow cross-country comparisons. Research covering multiple countries in a single study is strongly recommended. The observation of homogeneous built environment–infection associations in different geographic settings can strengthen the generalizability and causality of the results.

## 5. Conclusions

This review summarizes recent evidence regarding the associations between various built environment factors and SARS-CoV-2 infection risk. Areas with higher infection risks often feature dense commercial facilities, schools, and street networks, fewer green spaces, and accessible public transit. The evidence for some important built environment characteristics (e.g., urban density, green space, and land-use mixture) remains mixed. Understanding how built environment affects SARS-CoV-2 infection risk is critical to control the COVID-19 pandemic. This review provides valuable recommendations for policymakers and urban planners in post-pandemic planning and future urban planning practices. To address the barriers and limitations in the literature, future studies should use a longitudinal research design, focus on long-term effects, accurately measure both infection risk and built environment exposure, and cover diverse regions.

## Figures and Tables

**Table 1:** Associations between built environmental characteristics and COVID-19 outcomes among all 25 selected studies.

No.	References	Urban Density	Land-Use Mixture	Availability of GREENSPACE	Road Density	Accessibility to Public Transit	Accessibility to Intercity Train Station	Density of Commercial Facilities	Density of Hospitals	Density of Schools
1	Li, Peng [30]	0						+	0	
2	Yip, Huang [31]	+/0				0/0		+/+/+/0	0/0	
3	Credit [32]	−							0	
4	DiMaggio, Klein [33]	+/+								0
5	Hamidi, Sabouri [34]	+/0								
6	Gaskin, Zare [35]	0					0			
7	Hu, Yue [36]				+					
8	Huang, Kwan [37]	−/+/+	-			+				
9	Jin, Leng [38]					+		+/+/+/+/+	+	+
10	Johnson, Hordley [39]	0		−						
11	Klompmaker, Hart [40]			−						
12	Li, Zhou [41]	0	0		0	0		+	+	
13	Liu [42]	−/0		−		+				
14	Hamidi, Ewing [43]	-								
15	Ibrahim, Mohammed Eid [28]	0								
16	Nguyen, Huang [44]		+							
17	Rahman, Zafri [45]				0					
18	Scarpone, Brinkmann [46]						0			
19	Ma, Li [47]				+			+	+	+
20	You, Wu [48]	+		+					-	
21	You and Pan [49]	0		−						
22	Li, Ma [50]	+					+			
23	Tribby and Hartmann [51]	−		0						
24	Kan, Kwan [52]	0		+				−		
25	Sy, White [53]	0								

**Table 2:** Summary of associations between built environmental factors and coronavirus disease 2019 outcomes.

Built Environment Factors	Positive (+)	Negative (−)	Inconclusive (0)
Factors with strong evidence			
Commercial facility density (+)	11	0	2
School density (+)	2	0	1
Road density (+)	2	0	2
Accessibility to public transit (+)	3	0	3
Availability of green space (−)	2	4	1
Factors with weak evidence			
Urban density	8	5	11
Hospital density	3	1	4
Land-use mixture	1	1	1
Accessibility to intercity train stations	1	0	2

Note: Built environment factors with strong evidence are marked with a “+” or “−” in parentheses to show the direction of the association. Strong evidence means that the number of positive associations was greater than or equal to the total amount of negative or inconclusive associations, or negative associations was greater than or equal to the total amount of positive or inconclusive ones.

## Data Availability

The data presented in this study are available on request from the corresponding author.

## Appendix A

**Table A1:** Summary of study sample characteristics, study design, built environmental metrics, COVID-19 metrics, data analysis method, and results.

No.	References	Country	Study Design	Geographical Unit	Sample Size (*n*)	COVID-19 Metrics	Built Environment Metrics	Data Analysis	Results
1	Li, Peng [30]	China	Cross-sectional	1000 m buffer of COVID-19 cluster	639	Cluster size	1. Building density 2. Number of commercial facilities 3. Number of medical services	Structural equation model	Commercial vitality has a significant impact on the number of confirmed cases in an infectious cluster.
2	Yip, Huang [31]	China	Cross-sectional	Town Planning Unit (TPU)	154	Case number	1. Distance/number to clinics 2. Distance/number to restaurants 3. Distance/number to markets 4. Distance/number to metro stations 5. Population density	1. Cox proportional hazards regression 2. Ordinary least squares 3. Negative binomial regression	1.The data is divided into two phases before (Phase 1) and during the social distancing measure was relaxed (Phase 2). In Phase 1, clinics and restaurants are more likely to influence the prevalence of COVID-19. In Phase 2, public market, public transportation, and the clinics influence the prevalence of COVID-19. 2. In Phase 1, the areas of tertiary planning units with more restaurants are found to be positively associated with the period of the prevalence of COVID-19. In Phase 2, restaurants and public markets induce long time occurrence of the COVID-19. 3.In Phase 1, restaurant and public markets are the two built environments that influence the number of COVID-19 confirmed cases. In Phase 2, the number of restaurants is positively related to the number of COVID-19 reported cases.
3	Credit [32]	US	Cross-sectional	Zip code	-	Incidence	1. Population density 2. Hospital accessibility score	Ordinary least squares	Population density is negatively associated with COVID-19 infection rates at the neighbourhood-level.
4	DiMaggio, Klein [33]	US	Cross-sectional	Zip code	177	Case number	1. Population density 2. Housing density 3. School density	Bayesian hierarchical Poisson spatial models	Risk was approximately doubled by environmental characteristics such as population and housing density.
5	Hamidi, Sabouri [34]	US	Cross-sectional	County	913	Incidence	Activity density (population + employment)	Structural equation model	After controlling for metropolitan population, county density is not significantly related to the infection rate.
6	Gaskin, Zare [35]	US	Cross-sectional	County	3132	Case number	1. Population density 2. Distance/number to airports 3. Number of train stations	1. Negative binomial regressions 2. Cox regressions	The number of COVID-19 cases is positively related to proximity to airports.
7	Hu, Yue [36]	US	Cross-sectional	Town	357	Incidence	Road density	1. Spatial lag model 2. Spatial error model	Road density is significant explanatory variables.
8	Huang, Kwan [37]	China	Cross-sectional	TPU	291	Incidence	1. Population density 2. Residential density 3. Building height 4. Transport facility density 5. Land-use diversity	1. Global Poisson regression 2. Geographically weighted Poisson regression	1. Private residential density, transport facility density and building height have positive association with COVID-19 incidence. 2. Population density and land-use diversity have negative association with COVID-19 incidence.
9	Jin, Leng [38]	China	Cross-sectional with case-control	Neighbourhood	4329 (experiment) 17,316 (control)	Case number	1. Number of restaurants 2. Number of shopping centres 3. Number of hotels 4. Number of living facilities 5. Number of recreational facilities 6. Number of public transits 7. Number of educational institutions 8. Number of health service facilities	Multivariable logistic regression models	1. Having more restaurants, shopping centres, hotels, living facilities, recreational facilities, public transits, educational institutions, and health service facilities was associated with significantly higher odds of having COVID-19 cases in a neighbourhood. 2. The associations for restaurants, hotels, recreational, and education facilities were more pronounced in cities with fewer than six million people than those in larger cities.
10	Johnson, Hordley [39]	England	Cross-sectional	Local authority	299	Incidence	1. Availability of greenspace 2. Population density	Linear mixed effect models	After accounting for known mechanisms behind transmission rates, we found that park use decreased residual pre-peak case rates, especially when greenspace was low and contiguous.
11	Klompmaker, Hart [40]	US	Cross-sectional	County	3089	Incidence	NDVI	Negative binomial mixed models	1. An increase of 0.1 in NDVI was associated with a 6% decrease in COVID-19 incidence rate. 2. Associations with COVID-19 incidence were stronger in counties with high population density and in counties with stay-at-home orders.
12	Li, Zhou [41]	China	Cross-sectional	Community	1025	Incidence	1. Hospital density 2. Commercial facility density 3. Subway station density 4. Land-use mixture 5. Road density 6. FAR	1. Ordinary least squares model 2. Geographically weighted regression model	1. The distribution and density of major hospitals exerted a positive association with the epidemic situation. 2. The density of commercial facilities was the most prevalently distributed factor over the city that presented a positive association with the epidemic severity.
13	Liu [42]	China	Cross-sectional	City	312	Case number	1. Subway lines length 2. Per capita greenspace 3. Population density	Ordinary least squares model	1. Subway was positively connected with the virus transmission. 2. Population density was negatively associated with the spread of COVID-19 at the early stage of the epidemic.
14	Hamidi, Ewing [43]	US	Longitudinal	County	1165	Incidence	Population density	Multilevel Linear Model	After controlling for metropolitan size and other confounding variables, county density leads to significantly lower infection rates.
15	Ibrahim, Mohammed Eid [28]	Multiple conutries	Cross-sectional	Country	50	Case number	Population density	Stringency index model	Population density was found to be not a significant contributor in controlling COVID-19 epidemic in the very first month of spread.
16	Nguyen, Huang [44]	US	Cross-sectional	Zip code	30556	Case number	Land-use mixture	Poisson regression models	Indicators of mixed land use was connected with higher COVID-19 cases.
17	Rahman, Zafri [45]	Bangladesh	Cross-sectional	District	-	Incidence	Road density	1. Ordinary least squares model 2. Spatial lag model 3. Spatial error model4. Geographically weighted regression model	Road density had no significant influence on the occurrence rates of COVID-19.
18	Scarpone, Brinkmann [46]	Germany	Cross-sectional	County	401	Incidence	Access to long-distance train stations	1. Geospatial analysis heuristic 2. Geographical interpretation 3. Bayesian machine learning analysis 4. Generalised Additive Model	There appeared to be no significant observable partial dependence for long-distance train stations.
19	Ma, Li [47]	China	Cross-sectional	Town	2994	Incidence	1. Density of elementary and middle schools 2. Commercial facility density 3. Density of road intersections 4. Hospital density	1. Random forest approach 2. Bivariate local indicators of spatial association	The density of convenience shops, supermarkets, and shopping malls was one of the most important factors to infection cases.
20	You, Wu [48]	China	Cross-sectional	District	13	Incidence	1. Population density 2. Public green space density 3. Hospital density	1. Pearson correlation analysis 2. Spatial lag model 3. Spatial lag model	1. Increasing population density and public green space density were associated with an increased COVID-19 morbidity rate. 2. Increasing hospital density was associated with a decreased COVID-19 morbidity rate.
21	You and Pan [49]	US	Cross-sectional	County	989	Case number	1. Percentage of Urban Vegetation 2. Population density	Path analysis model	Each 1% increase in the percentage of urban vegetation will lead to a 2.6% decrease in cumulative COVID-19 cases.
22	Li, Ma [50]	China	Cross-sectional	City	255	Incidence	1. Centrality of railway stations 2. Population density	Mixed geographically weighted regression model	The associations are positive for the density of the POIs around railway stations.
23	Tribby and Hartmann [51]	England	Cross-sectional	ZIP code	144	Incidence	1. Population density 2. Density of park	1. Ordinary least squares model 2. Geographically weighted regression model	Population per square kilometre was negatively associated with case rates.
24	Kan, Kwan [52]	China	Cross-sectional	Large Street Block Group	1622	Case number	1. Density of commercial land 2. NDVI 3. Population density	Space-time scan statistic	More green spaces and lower commercial land density are linked to a higher risk for the residences of confirmed cases.
25	Sy, White [53]	US	Cross-sectional	County	1151	Basic reproductive number	Population density	Linear mixed models	Counties with greater population density have greater rates of transmission of SARS-CoV-2, likely due to increased contact rates in areas with greater density.
